# Supplementary material for: Novel Activity of ODZ10117, a STAT3 Inhibitor, for Regulation of NLRP3 Inflammasome Activation
Source: Int J Mol Sci. 2023 Mar 23;24(7):6079. doi: 10.3390/ijms24076079 (PMC10094431; doi:10.3390/ijms24076079)
Supplement: Supplementary file 1 [file ijms-24-06079-s001.zip › ijms-2232505-supplementary.pdf]

Supplementary Figures

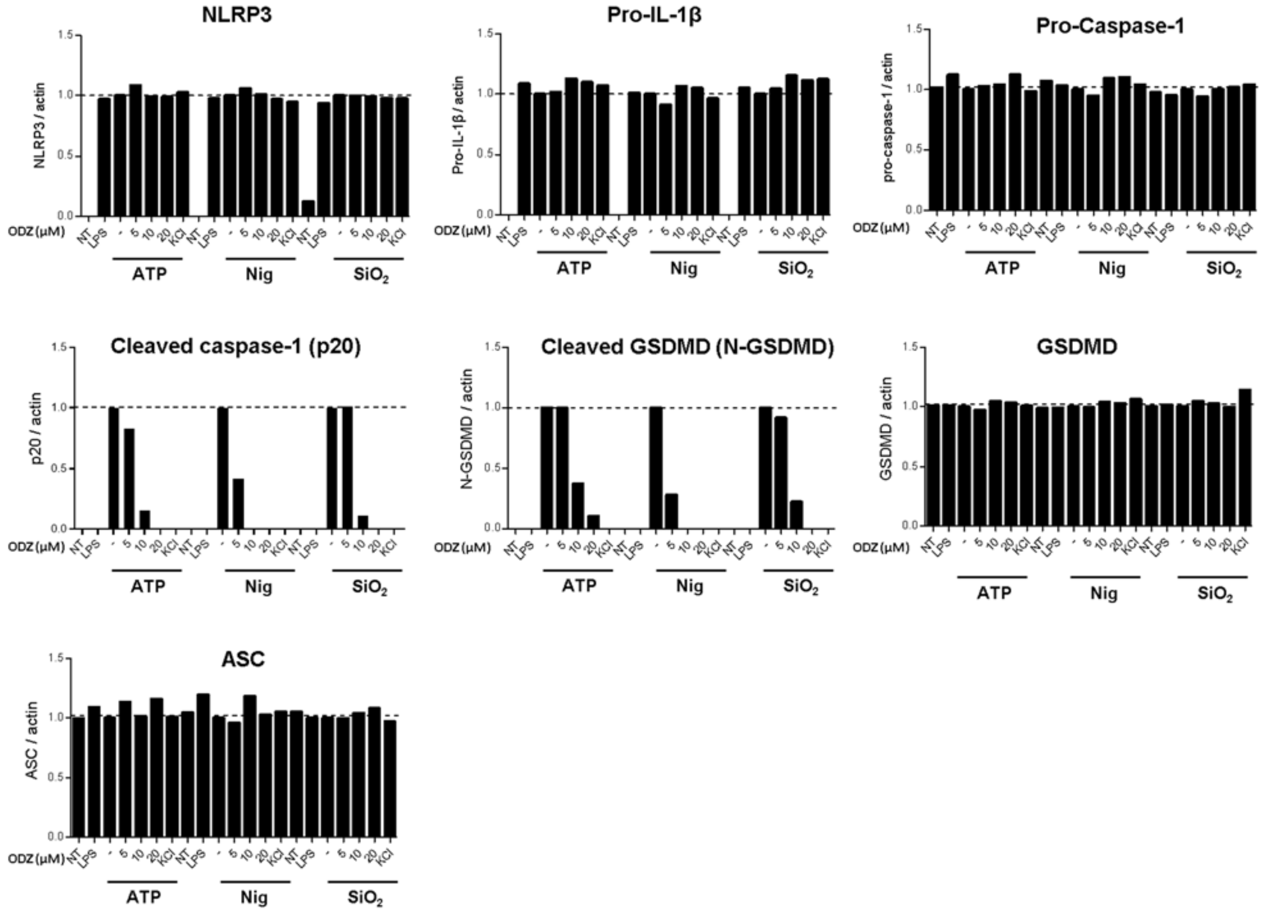

Figure S1. Densitometry analysis on the western blotting bands for the quantification of the data of Fig. 2. Densitometry analysis on the bands was performed using the NIH image J software and normalizing the data to total protein levels. β-actin served as an internal control.

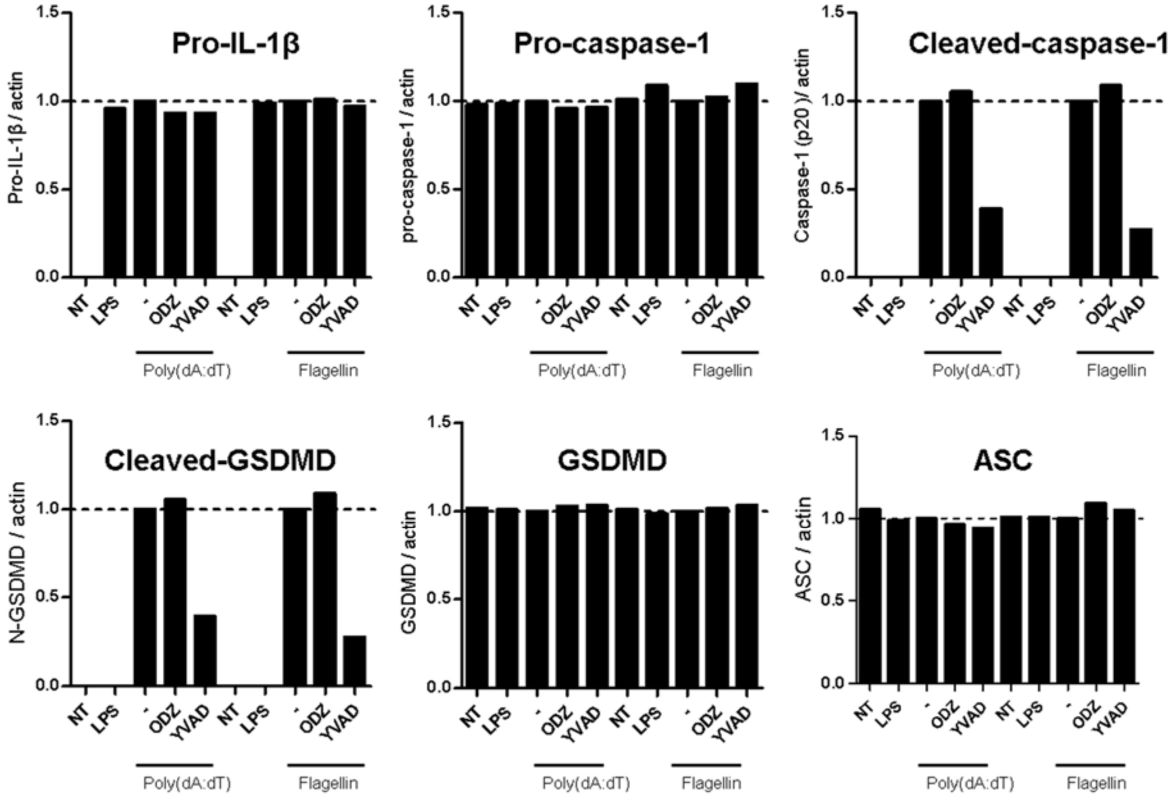

Figure S2. Densitometry analysis on the western blotting bands for the quantification of the data of Fig. 3C and 3D. Densitometry analysis on the bands was performed using the NIH image J software and normalizing the data to total protein levels. β-actin served as an internal control.

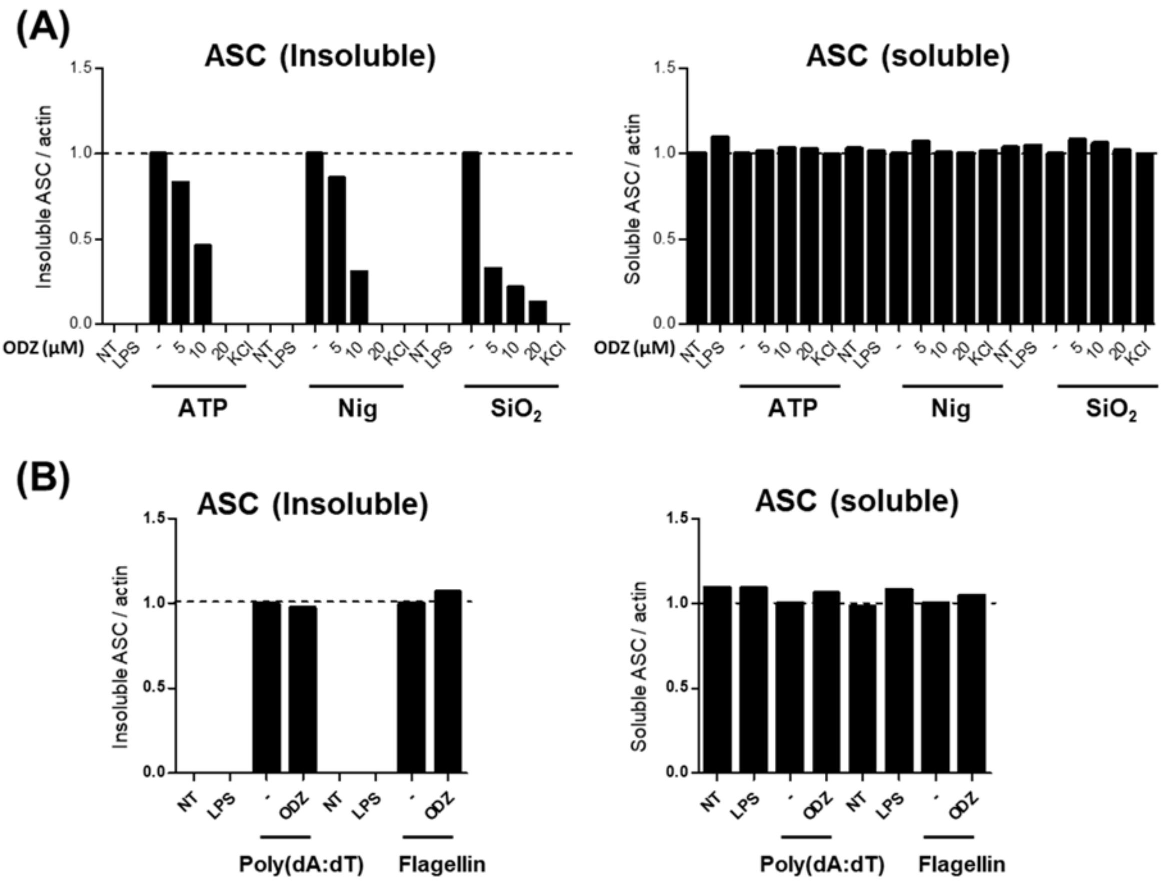

Figure S3. Densitometry analysis on the western blotting bands. (A) corresponds to Fig.4A and (B) corresponds to Fig.4E.

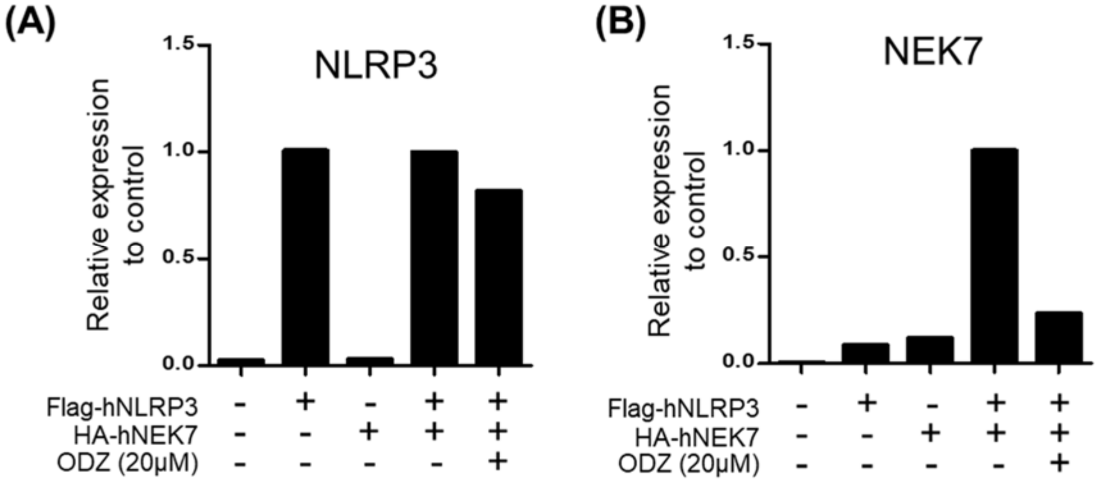

Figure S4. Densitometry analysis on the western blotting bands for the quantification of the data of Fig. 5.

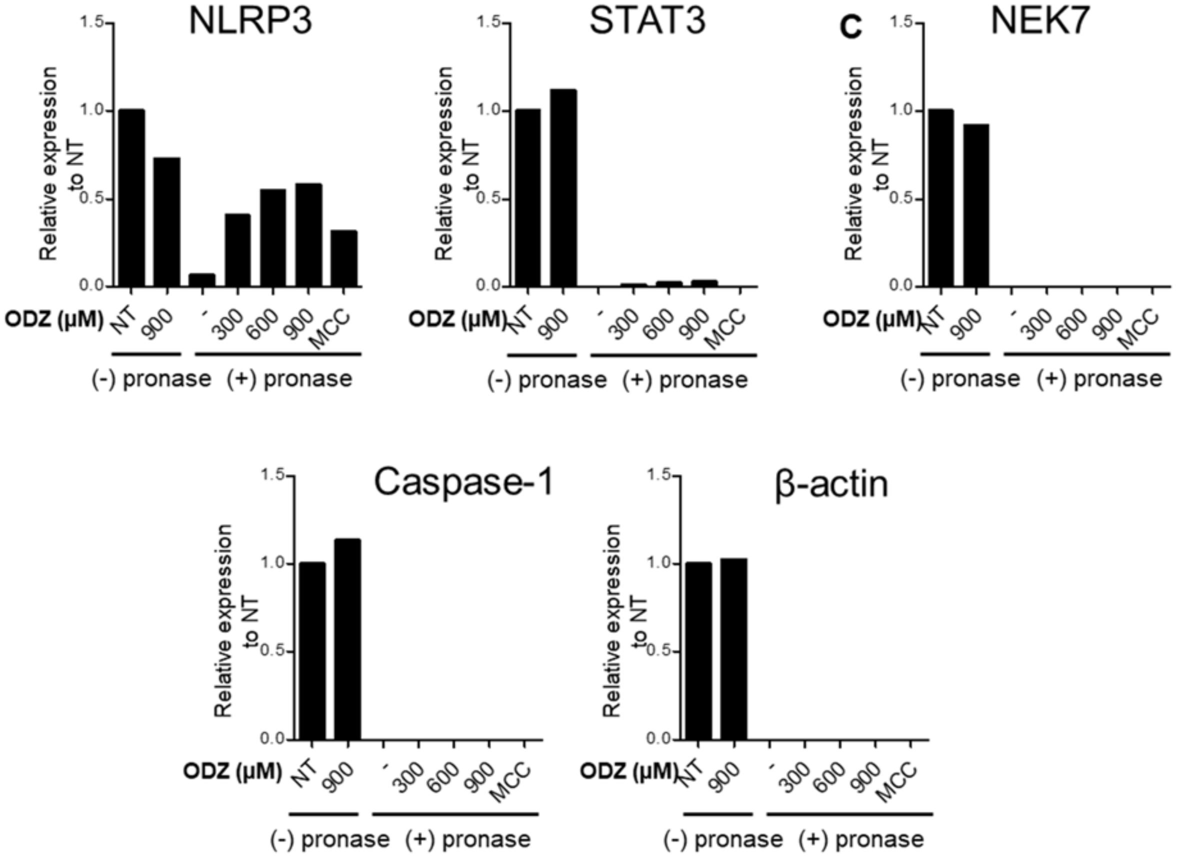

Figure S5. Densitometry analysis on the western blotting bands for the quantification of the data of Fig.6.
